# Combining incidence and demographic modelling approaches to evaluate metapopulation parameters for an endangered riparian plant

**DOI:** 10.1093/aobpla/plw044

**Published:** 2016-07-11

**Authors:** Noah D. Charney, Sydne Record

**Affiliations:** Department of Biology, Bryn Mawr College, 101 North Merion Avenue, Bryn Mawr, PA 19010, USA

**Keywords:** Colonization, extinction, Furbish’s lousewort, metapopulation model, occupancy, *Pedicularis furbishiae*, Saint John River

## Abstract

Metapopulations are a central concept in ecology and conservation biology; however, estimating key parameters such as colonization rates presents a substantial obstacle to modelling metapopulations in many species. We develop spatial and non-spatial simulation models that combine incidence- and demographic-based approaches to build a relationship between observed patch occupancy, habitat turnover rates, colonization rates and dispersal scales. Applying these models to long-term observations of *Pedicularis furbishiae* (Furbish’s lousewort), a rare plant endemic to the Saint John River, we predict that observed habitat patches averaging 550 m in length receive colonizing seedlings with a yearly probability of 0.45 or 0.54, based on two different models. Predictions are consistent with a standard analytic metapopulation formulation modified to partition extinction drivers during the early and the late phases of a population’s life cycle. While the specific results rest on several simplifying assumptions, the models allow us to understand the impact that increasing rates of habitat turnover would have on the future survival of this species.

## Introduction

Metapopulation theory is often used in quantitative conservation biology of rare species (Levins 1969; Morris and Doak 2002). Metapopulations can take various forms, including classic metapopulation models in which all sub-populations are similar and mutually dependent upon each other for recolonization following extinction (Levins 1969), source-sink models in which populations vary in their productivity (Pulliam 1988), and mainland-island models in which smaller populations experience a continual propagule rain from an external, extinction-immune source (Gotelli 1991). An important feature of metapopulations is that the processes governing local and regional population dynamics are fundamentally different from each other. Thus, patchy populations, in which patches are continuously connected by frequent dispersal, are not generally considered as metapopulations (Freckleton and Watkinson 2002). Before proceeding with the construction of a metapopulation model or basing management decisions on metapopulation dynamics, it is important to justify the reliance of a species’ viability on metapopulation dynamics, rather than local dynamics (Hanski 1995; Fronhofer *et al.* 2012). However, obtaining the parameters necessary (e.g. extinction, dispersal and colonization) to evaluate the metapopulation concept or parameterize such a model often proves prohibitively difficult, and thus the appropriateness of applying metapopulation dynamics to certain taxa, such as plants, has been questioned (Husband and Barrett 1996; Freckleton and Watkinson 2002). To estimate metapopulation parameters such as colonization, researchers have used various approaches, including direct observations with field data (Jäkäläniemi *et al.* 2005; Moody-Weis *et al.* 2008; Alexander *et al.* 2012), genetic distance metrics (Tero *et al.* 2003; Alsos *et al.* 2007; Robinson *et al.* 2013), mechanistic models (Nathan *et al.* 2001; Schurr *et al.* 2005) and estimates derived from simulations (Verheyen *et al.* 2004).

Models invariably make simplifying assumptions in order to make use of the limited data available in real-world systems and to strike a balance between parsimony and complexity (Levins 1966). Incidence-function models are attractive because they require only data on current patch-occupancy rates (Hanski 1994). However, they do not take into account demographic dynamics that occur within patches, they require data on dispersal distances, and they assume a stable configuration of habitat patches on the landscape (Akçakaya and Sjögren-Gulve 2000; Higgins and Cain 2002). The fact that the habitat itself is dynamic is increasingly recognized as one of the most important factors in predicting species’ fates (Thomas 1994; Wu and Levin 1994; Stelter *et al.* 1997; Ellner and Fussmann 2003; Snäll *et al.* 2003; Cornell and Ovaskainen 2008).

Here, we develop models that combines incidence- and demographic-based approaches to estimate colonization rates from long-term data on *Pedicularis furbishiae* (Furbish’s lousewort) while accounting for changes in habitat. We explicitly model habitat turnover and demographic extinctions separately in both a spatial and a non-spatial context that allows us to fit observed patch occupancy and to estimate colonization and dispersal rates. Our approach is similar to that of Verheyen *et al.* (2004), who modify an incidence function model to include habitat turnover and colonization rates for forest plants in Lincolnshire, UK over the past several hundred years. In this paper, we extend the framework of Verheyen et al. (2004) for estimating colonization rates and apply it to an endangered riparian plant, *P. furbishiae*. Our specific research objective is to calculate a first-order approximation for the colonization rates of *P. furbishiae.*

## Methods

*Pedicularis furbishiae* (Furbish’s lousewort) is endemic to a 225-km stretch of the Saint John River on the border of Maine and New Brunswick. It was one of the first species listed under the US Federal Endangered Species Act, it was the subject of one of the first published population viability analyses on a plant species, and all known sub-populations have been regularly monitored since 1976 (Menges 1990). The bank habitat of *P. furbishiae* exists in a tenuous dynamic between erosion and succession (Gawler *et al.* 1987). Bank collapse and ice scour along the river can rapidly wipe out entire sub-populations. However, when no erosion occurs, *P. furbishiae* will be out-competed by later successional species. Because it appears that any individual habitat patch will remain suitable only for a limited period of time, metapopulation dynamics are thought to play an important role in the species’ persistence (Menges 1990).

Field data on habitat turnover and patch occupancy were obtained from the Maine Natural Areas Program that oversees the monitoring and recovery of this species. Between 1976 and 2008, 35 *P. furbishiae* patches were monitored for a total of 957 patch years. Stem counts of flowering individuals were conducted on 13 occasions over this period. In this time, four patch extinctions were observed due to either erosion or competition (D. Cameron, Maine Natural Areas Program, personal communication). Because only extant sub-populations were continuously monitored, there is no information on colonization events.

**Figure 1.**
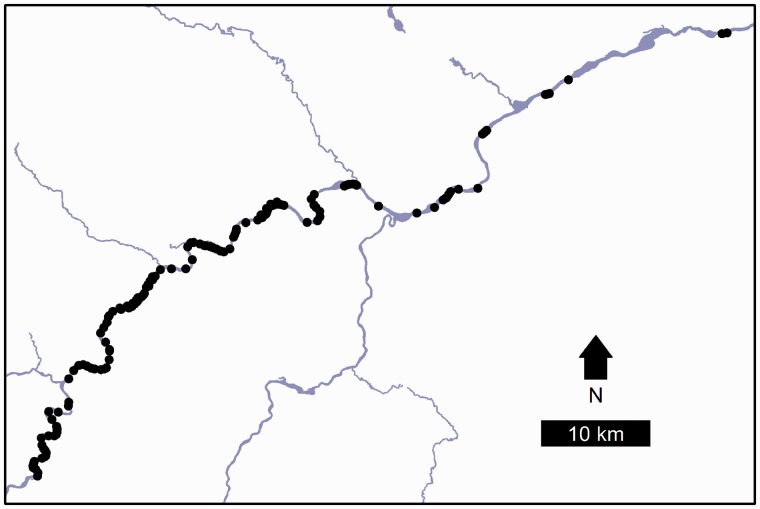
Map of patches surveyed for potential *Pedicularis furbishiae* habitat along the Saint John River in Maine, USA. Points represent the 149 patches of suitable and unsuitable habitat used in our simulations.

In 2008, the Maine Natural Areas Program mapped available habitat surrounding known populations of *P. furbishiae* (Fig. 1). To determine habitat suitability, expert opinion based upon slope and existing vegetation was used to classify river stretches as either ‘potential *P. furbishiae* habitat’, ‘too dry’, ‘too steep’ or ‘too flat’. Out of 72 km of river bank surveyed along a 100-km stretch of river, 35 km was identified as suitable habitat. The suitable habitat was composed of 61 discrete patches, of which 47 contained extant populations of *P. furbishiae*. There were two additional patches with suitable habitat for which it was not clear from the provided field notes whether or not *P. furbishiae* were surveyed for or detected in these patches, and so we excluded these patches when calculating the current occupancy rates. We defined habitat patches based on current contiguous areas of suitable habitat bounded by areas of unsuitable habitat. The mean length of the suitable habitat patches as measured along the river was 550 m (SD = 560 m, min = 29 m, max = 2436 m). These data also include 86 patches of unsuitable habitat, with a mean length of 410 m (SD = 390, min = 32, max = 1707).

We developed two simulation models for the *P. furbishiae* metapopulation: a non-spatial model and a spatial model. Both of these models are hierarchical, in which demographic processes play out within envelopes of simulated habitat processes. Patches switch between suitable habitat and unsuitable habitat based on simple random probabilities. These habitats may then be colonized by seedlings, which grow and reproduce according to a stochastic stage-based demographic model. With the non-spatial model, we use simulated past conditions to understand what values of metapopulation parameters would give rise to the currently observed conditions. With the spatial model, we used the current conditions as a starting point and examined the effect of metapopulation parameters on simulated future dynamics. All models were constructed in R Statistical Software (versions 3.1.0 and 3.2.0), and are provided along with detailed instructions on their use in the supplemental documents associated with this manuscript [**see Supporting Information**].

### Non-spatial model

Our non-spatial model relates three primary parameters which we treated as constants: colonization probability, habitat failure probability and patch occupancy. We use this framework to ask what combination of historic habitat failure and historic colonization rates would yield the observed current patch occupancy. The central concept in this model is to treat habitats as if each has a life span with a discrete beginning year and a discrete ending year [**see Supporting Information – Fig. 1**]. Any given patch can only contain a single habitat at any time. We define a habitat’s age as how many consecutive years the focal patch has contained habitat capable of supporting any plants or seeds (suitable habitat) prior to the present. We expect that the habitats observed in our 2008 sampling year represent a distribution of habitat ages. For instance, in a patch containing a habitat with an age of 100 years, there would have been continual suitable habitat in that patch every year from 1908 until 2008, but there would not have been any suitable habitat within that patch in 1907. Any habitat that may have existed prior to 1907 in the focal patch is not considered to be part of the same continual habitat, as, by definition, all plants and seeds would have been eliminated from that patch in 1907.

We considered patch occupancy to be the number of habitat patches that contain a population detectable by field surveys. In our case, a population would need to have had at least one plant in the reproductive stage to be detectable since the yearly monitoring focused on stem counts of flowering individuals. The colonization probability for a focal habitat patch was the annual probability that a single seed from outside the patch would arrive at the patch and germinate. By treating colonization probability as fixed across space and time, our non-spatial model does not incorporate the dispersal of individuals from patches. Rather we only model individuals coming into patches. We defined habitat failure as the annual probability that all plants and seeds in a given patch would be completely eliminated by changes in habitat quality (e.g. ice scour, succession). Habitat failure does not include sub-population extinction due to demographic stochasticity, which is modelled separately with the stage-based matrix approach.

For this study, we treated all habitat patches equivalently, we do not incorporate explicit spatial information (e.g. distance between patches, patch size) and we treated the colonization and failure probabilities as fixed across time and space. The trajectories of plants in individual patches were tracked separately with all patches governed by the same global parameters (colonization probability, habitat failure probability, the set of demographic transition matrices and carrying capacity), rather than separate site-specific parameters.

**Figure 2.**
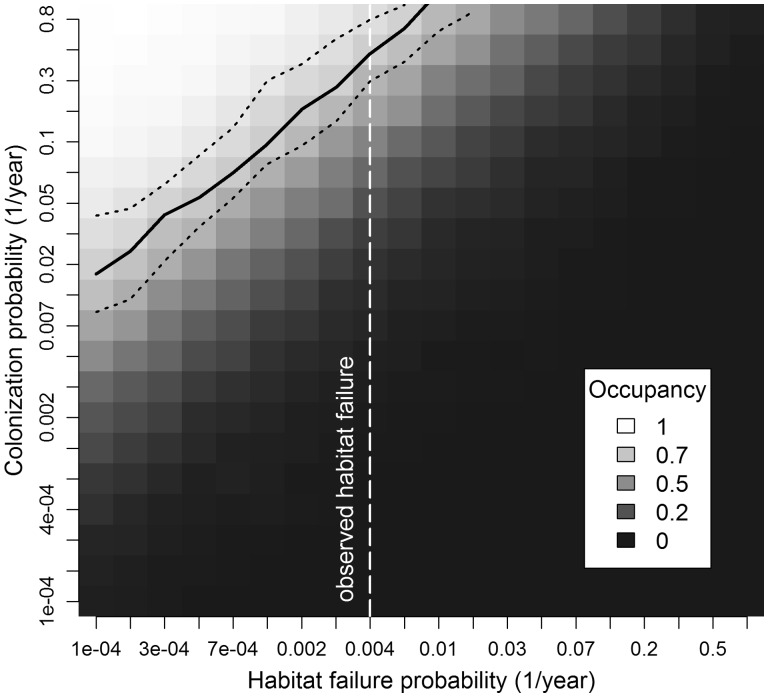
Simulated occupancy in a non-spatial model of *Pedicularis furbishiae* at 61 habitat patches estimated from 15 iterations at each parameterization of colonization probability and habitat failure probability. Patch occupancy is defined as the number of habitat patches with at least one reproductive adult. Colonization probability is the yearly probability for a given unoccupied habitat patch that a seedling will establish in that patch. Habitat failure probability is the yearly probability that a catastrophic event will eliminate all plants in a given habitat patch.

In executing the non-spatial model, we: (i) simulated habitat ages for each of our 61 observed habitat patches, (ii) simulated historic colonization and growth within these patches over the lifetime of all patches ending in 2008 and (iii) compared the ending simulated occupancy with observed occupancy in 2008 [**see Supporting Information – Fig. 2**]. In each iteration, we first used the habitat failure probability to generate a distribution of habitat ages for the modelled habitats by drawing randomly from an exponential distribution. We chose an exponential distribution to reflect the fact that all habitat patches have an equal failure probability in every year. The habitat age structure formed the envelope within which stage-based matrix population projections occurred, beginning from the age of the oldest simulated habitat. Because the non-spatial model does not include emigration of seeds from patches, there is no reason to model plants within habitats that do not currently exist. In the example of a 100-year old habitat, we know that the focal patch contained zero plants in 1907, and so we begin modelling habitat within that patch in 1908. In the first year of our simulation, colonizing seedlings of *P. furbishiae* could only survive within the oldest habitat, because no other modelled habitats were yet in existence. As the years progressed, more patches were incorporated according to the random age distribution until 2008 when all 61 patches contained suitable habitat. Because colonization probability remains constant throughout the simulations, and all simulations begin with no plants or seeds in any patches, it is implied that there are other populations outside of our model which provide a seed source.

**Figure 3.**
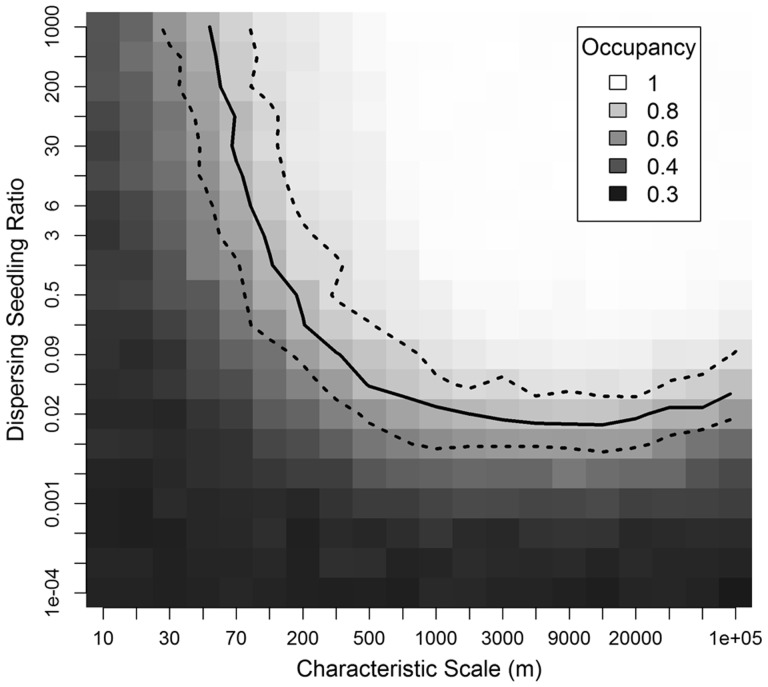
Simulated occupancy in a spatial model of *Pedicularis furbishiae* at 149 patches estimated from 20 iterations at each parameterization of characteristic scale and dispersal ratio. Patch occupancy is defined as the number of patches with at least one reproductive adult. The characteristic scale defines the exponential distribution used for seedling dispersal. The dispersal ratio specifies the ratio of additional dispersing seedlings relative to the number of seedlings recruited within each patch.

In each time step, each patch with suitable habitat received one extra seedling with a probability as determined by the colonization probability. Plants then grew according to a stage-based matrix projection model parameterized by Gawler (1988). These models contain six stages (seedling, juvenile, vegetative, small flowering, medium flowering and large flowering [**see Supporting Information – Fig. 3**]). Gawler monitored individual plants within 13 plots for 3 years to create 26 separate transition matrices. We incorporated stochasticity into our model by resampling from the 26 transition matrices using a multinomial distribution for transitions and a log normal distribution for fertilities with the package ‘popbio’ (Stubben and Milligan 2007) in the statistical software R version 2.11.1 (R Development Core Team 2010). We set carrying capacity for each population at 1500 plants, which is the approximate maximum observed population size. At the end of each iteration, we calculated patch occupancy as the proportion of habitat patches in the final year containing at least one reproductive plant.

**Figure 4.**
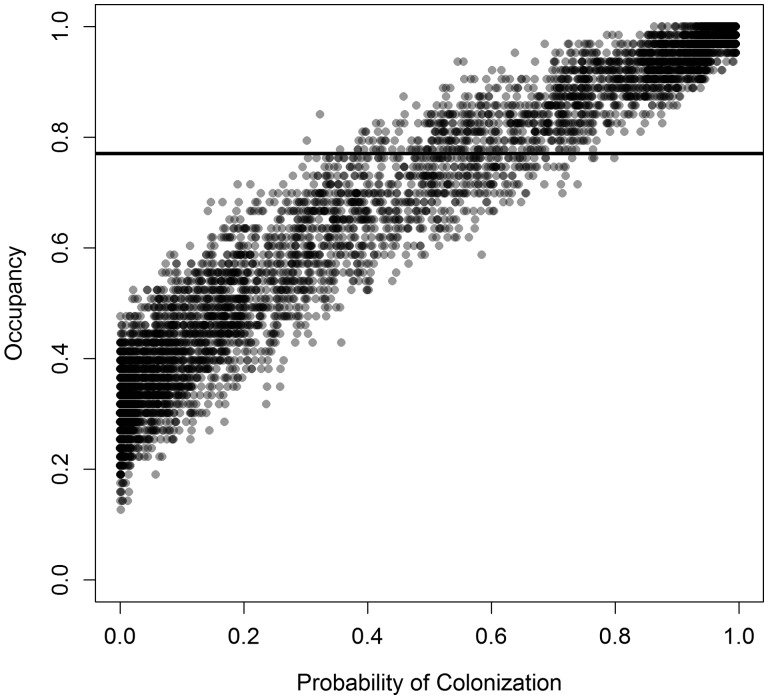
Simulated occupancy in a spatial model of *Pedicularis furbishiae* at 149 patches versus the probability that a patch was colonized by one or more seedlings in a given year for the 8000 model runs represented in Fig. 3. Colonization probability is averaged across patches and years for each run. The horizontal line represents the observed occupancy. Points are semi-transparent so that darker areas represent more simulations.

We varied both habitat failure probability and colonization probability on a log scale between 0.0001 and 1, with 20 steps for each parameter. At each of the 400 parameterizations, we calculated the mean patch occupancy from 15 iterations, for a total of 6000 model runs. We calculated the upper and lower 95 % confidence intervals for each parameterization by fitting a beta-distribution to the distribution of 15 occupancies in each iteration and then calculating the central 95 % of the fitted beta-distribution. In preliminary tests, we found that estimating confidence intervals with this method from 15 iterations did a good job of matching the confidence intervals derived from 1000 iterations while reducing computational overhead [**see Supporting Information – Fig. 4**].

We then matched the simulation outputs with the observed habitat failure rates and observed patch occupancies to obtain an estimate for the colonization probability. With an estimate of the total metapopulation size and seed set for *P. furbishiae*, we translated colonization probability experienced by any patch, modelled as a binomial distribution, into the dispersal probability for a given seed.

### Spatial model

The basic framework of the spatial model is the same as the non-spatial model: sub-populations grow according to a stage-based demographic model within habitat patches that have a global probability of failing in any given year. The core differences here are that: (i) we allow patches to switch back and forth between habitat and non-habitat, (ii) we explicitly model dispersal events between patches in a spatial framework and (iii) not all patches have the same carrying capacity.

We begin each spatial simulation based on linear features in 2008 GIS data provided to us by the Maine Natural Areas Program. We condensed these data into two spatial metrics used to describe each patch in our model: position of patch centre and length of patch along river. These patches are not all contiguous, as there are many stretches of river bank between these patches that were not surveyed. We do not model intervening patches that were not observed.

The initial abundances in the spatial model reflect estimated sub-population sizes based on available population surveys. These surveys do not cover all sub-populations, only contain flowering stem counts, and generalize across broad stretches of river without detailed locality information. Based on these surveys, the Maine Natural Areas Program estimated the global population to be ∼2100 flowering stems in 2008. We thus distributed flowering stems amongst the patches where surveys locality data could be matched to our patch data. We then used a Poisson distribution to randomly distribute the remainder of the flowering stem population amongst the patches with known current occupancies, but no detailed stem counts. We used the average of our 26 transition matrices to calculate average stable stage distributions, and populated each patch with individuals at every stage to match both the number of flowering stems assigned and the stable distribution. This was necessary as the field survey only counted flowering stems. We generated this initial distribution of plants across the metapopulation once, and used it as the starting condition for all iterations of the spatial model.

In each year, we allowed patches to randomly switch from suitable to unsuitable habitat based on the observed habitat failure rate. We then replaced these failed habitats by randomly selecting unsuitable habitat patches to become suitable habitat in order to maintain a constant number of available habitat patches. Thus, if 10 suitable habitat patches became unsuitable in a given year, we balanced this out by switching the habitat status of 10 of the previously unsuitable patches. Replacing failed habitats with new habitat allowed us to focus on the role of our dispersal parameters in maintaining equilibrium dynamics, which is the focus of this study. Otherwise, the simulations would have uninterestingly resulted in predictable declines in the global population as controlled by the number of available habitats remaining in the simulation.

Demographic growth occurred within each patch in the same manner as the non-spatial model: sampling randomly from the 26 transition matrices provided by Gawler (1988). We set carrying capacities of patches in direct proportion to patch length, with the largest patch carrying capacity equal to 1500 flowering stems, the approximate maximum observed population size, and smaller patches with proportionately smaller carrying capacities.

We simulated dispersal of seedlings in each time step after the demographic model ran for each patch. The dispersal process can be broken into three steps: (i) calculate the number of dispersing individuals leaving each patch, (ii) calculate all pairwise dispersal probabilities ($d_{ij}$) between patches and (iii) add each dispersing seedling to another patch (or outside of the modelled space) based on the calculated dispersal probabilities.

In simulating dispersal, to be consistent with our stage-based transition matrix, we modelled colonization of established seedlings, rather than movement of seeds *per se*. We treated dispersing individuals as additional recruitment occurring outside of the focal patch. We designated the parameter, *r*, as the ratio of additional recruitment outside of the patch boundaries relative to internal recruitment. For example, if *r* = 0.1, for every 10 seedlings established at the end of a demographic run within patch *i*, we would allow 1 additional seedling to disperse from patch *i* to another point along the river. We use this approach because fecundity estimates in these data are based on empirical observations of recruitment relative to local flowering density, rather than mechanistic models of germination, predation and herbivory (Menges 1990). Unlike mechanistic fecundity models, such empirical fecundity estimates would not include recruitment of seedlings that dispersed and established in other patches. Although colonizing seedlings establishing within a patch would be counted in empirical fecundity estimates, we expect the number of seedlings falling within a patch to be small relative to the number that leave the patch.

When allowing seedlings to disperse, we used a 1D spatial geometry reflecting the linear nature of rivers. We calculated the pairwise distances between all patch centres as measured along the path connecting the points. We considered four factors in this spatial process. First, considering that dispersal between close patches is more likely than dispersal between distant patches, we modelled dispersal probability based on the exponential distribution, $P=\;\tau e^{-\tau x}$ , where *P* is the probability density, *x* is the distance and 1/*τ* is the characteristic scale of the function. Secondly, because a larger patch makes a larger target that is more likely to receive dispersing individuals, we integrated *P* along the length of the potential receiving patch to calculate dispersal probability. Thirdly, we accounted for the fact that dispersal is more likely to occur downstream than upstream, with the parameter, *f*, which is the fraction of seedlings that disperse downstream compared with the total number of dispersing seedlings (upstream and downstream). Fourth, to account for the fact that it may be more difficult to disperse across the river to the opposite bank than to disperse to another patch on the same bank, we added an extra distance cost term *bτ*, prior to calculating dispersal probability between patches that were on opposite banks from each other. The final dispersal function is then,

dij=f∫x1x2τe-τxdx, where j is downstream                  from i on same bank1-f∫x1x2τe-τxdx, where j is upstream                      from i on same bankf∫x1x2τe-τ(x+bτ)dx, where j is downstream                     from i on opposite bank1-f∫x1x2τe-τx+bτdx, where j is upstream                     from i on opposite bank

where $d_{{}_{ij}}$ is the probability that a seedling dispersing from patch *i* will land in patch *j*, *f* is the fraction of dispersing seedlings that travel downstream, *x*_1_ is the distance between the centre of patch *i* and the near edge of patch *j*, *x*_2_ is the distance between the centre of patch *i* and the far edge of patch *j*, 1/*τ* is the characteristic scale and *b* is the cost factor for dispersing across the river. This dispersal function is only defined for seedlings that are already determined to be dispersing outside of their home patch—thus it does not include the possibility for a seedling to stay within the home patch. We do allow dispersing seedlings to land outside of our modelled patches, thus permanently leaving our simulation. The probability that an individual dispersing from patch *i* will not fall within one of our modelled patches is given by $1-\underset{j}{\sum }d_{ij}$.

In running our spatial model, we allowed the characteristic scale (1/*τ*) to vary between 10 m and 100 km, and we allowed the dispersal ratio (*r*), to vary from 1/10 000 to 1000, running 20 iterations at each parameterization for a total of 8000 model runs. During these trials, we fixed the fraction of downstream dispersal (*f*) at 0.9, and the cost factor for dispersing across the river (*b*) at 1. We performed a separate sensitivity analysis in which we allowed *b* to vary between 0.1 and 20 and *f* to vary between 0.01 and 0.99, while 1/*τ* was fixed at 200 m, and *r* was fixed at 0.04.

## Results

In our non-spatial model, the intersection of the observed annual habitat failure rate, 0.0042 (4 failures in 957 patch years), and the observed patch occupancy, 0.77, occurred in our model at an annual colonization probability of 0.45 (95 % CI = 0.29–0.75; Fig. 2). In this region, a 60 % increase in habitat failure probability results in a loss of ∼7 % of the occupied habitats at equilibrium. A 60 % increase in colonization probability in this region would result in a gain of ∼16 % more occupied habitats.

In the spatial model, the occupancy at the end of simulation runs was affected by both the characteristic scale and the number of dispersing seedlings. Within this parameter space, the ending occupancy matched our observed occupancy, 0.77, for simulations along a curved path (Fig. 3). When the dispersal scale was between 1 and 100 km, simulated occupancy matched the observed occupancy when the dispersal ratio was between 0.007 and 0.4. When the dispersal ratio was >1, the characteristic scale needed to be between 30 and 200 m for the simulation occupancy to match the observed occupancy. In 134 simulations for which simulated and observed occupancy matched, the mean annual probability of a patch receiving one or more dispersing seedlings was 0.54 (SD = 0.11; Fig. 4). Colonization was bi-modally distributed, with many patches receiving no dispersing seedlings and many patches receiving dispersing seedlings every year. In the 134 simulations matching observed occupancy, the mean annual probability of an unoccupied patch received dispersing individuals was 0.51 (SD = 0.11), whereas in occupied patches, this probability was 0.6 (SD = 0.13). Increasing the cost factor for dispersing across the river (*b*) caused ∼6.5 % lower occupancy rates [**see Supporting Information – Fig. 5**]. Compared with simulations with no downstream bias in seed dispersal, when *f* = 0.5, occupancy rates were 7–9 % lower when *f*  ≥ 0.99, and 13–14 % higher when *f * ≤ 0.01.

Using the 2008 estimated population size of *P. furbishiae* of 2100 flowering stems with an average of 60 viable seeds per stem the global pool of seeds each year would be ∼125 000 (Menges *et al.* 1986; Gawler 2008). With a colonization probability of 0.4 (from the non-spatial model) or 0.54 (from the spatial model), each year ∼24 or 33 of the 61 habitats would gain an established seedling that originated from a different patch. This equates to a minimum of ∼1 in every 5000 or 1 in every 3800 seeds dispersing to a different habitat patch and surviving to the seedling stage.

Extinctions of established populations were primarily driven by the catastrophic habitat failures within our models, not demographic extinctions. Considering all 8000 runs of the spatial model, in the patches that began the simulations with *P. furbishiae* populations but that subsequently experienced extinctions during the course of the simulations, 81 % (SD = 39 %) of these extinctions were caused by habitat failures. The percent of extinctions caused by habitat turnover ranged from an average of 60 % in parameterizations with few dispersing seedlings and small spatial scales to 100 % in parameterizations with many dispersing seedlings and large dispersal scales. Inspecting the 134 runs in which final occupancy most closely matched observed occupancy, extinction events of the initial populations were caused by habitat failures in 85 % (SD = 6 %) of the cases. In these 134 runs, the starting populations persisted on average for 150 years, with 50 % of the populations persisting all 200 years of the simulations. With an annual rate of extinction, *e*, one would expect the fraction of remaining populations, *y*, at time, *t*, to be *y* = (1 − *e*)^*t*^. Solving this equation, when 50 % of the populations remain at 200 years, the annual extinction rate would be 0.0035.

## Discussion

Our metapopulation approach combines a model of habitat dynamics with a demographic matrix model of population growth. Recognizing that colonization is a rare event that is difficult to observe (Husband and Barrett 1996), our framework estimates colonization by taking advantage of the types of data that are often available to researchers: demographic data and habitat data. *Pedicularis furbishiae* is a classic example of a metapopulation in the plant literature (Menges 1990). Yet, even with decades of study, it remains difficult to directly estimate rates of colonization.

The dynamics in our simulations conform to patch-tracking metapopulation models, such as those observed for epiphytes on trees by Snäll *et al.* (2005). In patch-tracking systems, habitat dynamics are more important than demographic stochasticity in determining a species’ trajectory. As an Endangered species in both the USA and Canada, *P. furbishiae* receives much interest from conservation agencies as well as the general public (NatureServe 2014). A primary focus of management for this species involves understanding and mitigating for the threat of eroding river banks. Managers are concerned about the potential for banks to become increasingly destabilized by development along the river margins and by more frequent flood events in a changing climate (D. Cameron Maine Natural Areas Program, personal communication). Our study provides a framework that managers can use to quantitatively translate projected changes in habitat failure rates into impacts on the entire *P. furbishiae* metapopulation.

The primary research objective of this study was to provide a first-order approximation of the colonization rate for the single global population of *P. furbishiae.* Our estimate of the colonization rate (∼0.5) is high relative to the habitat failure rate (0.004). The high colonization rate does not imply that new reproductive populations establish at this rate, as our definition of colonization includes any seeds that establish in a patch, not just those that survive to reproductive maturity. Given the low survival rate of young plants, in simulation trials we find that the probability of a single seedling growing and reproducing into a population that still exists 50 or 200 years after colonization is only 3.0 or 2.7 %, respectively. In contrast, the probability of a set of five mature plants producing a population that still exists in 50 or 200 years is 73 or 64 %, respectively. Thus, the rate at which populations establish from seedlings is relatively low; however, once reproducing populations are established, they typically grow and persist as dictated by the positive transition matrix growth rates (*λ*), as long as the habitat remains viable.

Comparing with analytic models, our non-spatial simulation is analogous to the simple ‘propagule rain’ metapopulation model (Gotelli 1991), in which the equilibrium occupancy ($\hat{P}$) is given by $\hat{P}=\frac{c}{c}+E\;\frac{}{}$, where *E* is the extinction rate and *C* is the colonization rate. However, this analytic model assumes that initial colonization events are equivalent to full population establishment. In our system, demographic stochasticity drives high extinction rates early in a population’s existence, but as the population matures, habitat turnover becomes the driving force in extinction. To account for this dynamic, we can make an approximate first-order adjustment to the analytic equation by considering the colonization term to include only the fraction of seedling colonization events that result in long-term population establishment, *s* ≈ 0.03. In this sense, *s* accounts for extinctions driven by demographic stochasticity early in a population’s life history, whereas *E* accounts for habitat-driven extinction once a population has reached a stable size. A modified equation might then be, $\hat{P}\approx \;sC/(sC+E)\frac{}{}$. Solving this equation with our observed occupancy and extinction rates yields an equilibrium colonization rate of *C* ≈ 0.45, which agrees well with our simulation results. This formulation is approximate, as it implies a time lag in the colonization–extinction process, without precise definitions for the population age at which *s* should be calculated, how to measure occupancy, and how extinction is defined. While a full treatment of this analytic formulation is beyond the scope of the current study, this cursory examination is sufficient to demonstrate that our results are reasonable given existing theory.

*Pedicularis furbishiae* is considered a classic example of a plant metapopulation; however, the relatively low rates of turnover which we report differ from what was previously reported for this species. The original metapopulation framework for this was based on field work by Gawler (1988) and Menges (1990) conducted at a smaller spatial and temporal extent than in our study. Compared with our analysis, Menges estimated that populations go extinct, and new populations are established at much higher rates, ranging from 0.02 to 0.125 per year. Here, Menges includes only individuals that reach reproductive maturity in his definition of colonization. Menges reports that 3 of 10 populations went extinct during the 6 years of the study, yet we observed extinctions in only 4 of 35 populations over a 32-year period. If our definition of patch size was much bigger than Menges’ definition, this could explain the difference in our estimates, as one would expect more frequent turnover at a fine grain within populations. However, it appears from the available data that the patch size definitions in the two studies are roughly equivalent. Based on Menges’ extinction rates ranging from 2 to 12.5 %, the expected age of any population would be between 8 and 50 years. This led Menges to conclude that ‘individual *P. furbishiae* populations are temporary features of the riverine ecosystem’ (Menges 1990). However, in our models, the mean expected population age is 289 years. Although *P. furbishiae* populations may not be absolutely permanent features on the landscape, we find that they are much less fleeting than was previously suggested. It seems that Gawler and Menges may have happened upon a particularly unstable set of populations in an especially unstable set of years, thus biasing turnover estimates. While colonization and extinction of entire populations might play an important role across centuries, our study suggests that these forces have been relatively rare events across the last few decades. It is important to understand the temporal scale of metapopulation dynamics, as managing a system in which sub-population life expectancy is only 8 years compared with a system with sub-population life expectancies close to 300 years may warrant a very different approach.

In our spatial model, the dispersal ratio (*r*) acts as an artificial boost to fecundity, because we add dispersing individuals to the existing pool of recruits. Thus, for values of *r* large relative to 1, we see high occupancy rates in the model. The alternative approach would have been to take dispersing individuals out of the total number of recruits based on our fecundity transition matrices. This would have resulted in artificially lowering fecundity, but would have been more appropriate had fecundity estimates been based on mechanistic models of recruitment.

Our models should be thought of as merely a first-order approximation for the parameters of colonization and extinction under equilibrium conditions. This study should not be taken as a population viability analysis, as we explicitly seek to simulate stable dynamics in which the global population neither decreases nor increases. Thus, every time a habitat failed in our spatial simulation, we replaced it with a new habitat, but this may not be the reality in the future. In order for us to obtain these estimates, we had to make several gross simplifications regarding the demography and occupancy of the species. In the non-spatial model, as with many incidence-based models, we assume that a dynamic equilibrium has been reached on the landscape (Guisan and Zimmerman 2000). This assumption is most problematic in the parameterizations where the habitat failure rate is very low. In the region of the non-spatial model where our observed parameters intersect, the model simulations reached equilibrium within ∼200 years. In regions with higher failure rates or higher colonization rates, equilibrium was reached much sooner. However, in regions with much lower habitat failure probabilities, equilibrium is not reached for thousands of years. Dynamics on the river may arguably have been fairly stable over the past few centuries, but certainly river dynamics have changed over the past few thousand years. In addition, the habitat patches which we have modelled likely do not correspond with population patches. Indeed, very long stretches of habitat may have multiple discrete sub-populations within them. Furthermore, long stretches of habitat likely experience multiple smaller habitat turnover events rather than the entire habitat failing at once.

There are several ways in which these models could be improved to better approximate the actual system dynamics, including adding in spatial autocorrelation, incorporating seed banks or exploring nested scales. Perhaps the biggest limitation is the fact that the demographic transition data were all collected from a set of just 4 years. This analysis remains a first approximation and additional monitoring, especially more detailed and longer-term demographic and continued occupancy studies, would help to improve upon this modelling exercise to better understand the metapopulation status of this species of conservation concern.

## Conclusion

This study illustrates an approach for estimating missing metapopulation parameters when critical data needed for understanding a species’ spatial and temporal patterns are unavailable, or impossible, to obtain. Such an approach may be of particular utility for rare and endangered species for which little population-level data exists. Non-spatial and spatial simulation models of the metapopulation dynamics of *P. furbishiae* result in similar estimates of the equilibrium colonization rate for this species that align with estimates based on analytic approximations from existing theory. Yet we must keep in mind that these remain highly simplified models of a plant whose true ecology is governed by many complex processes not captured herein.

## Sources of Funding

N.D.C. was funded by the National Science Foundation Graduate Research Fellowship Program. S.R. was funded by NSF DEB-0909604.

## Contributions by the Authors

N.D.C. conceived of and coded the models. S.R. helped with model development and data acquisition. Both authors contributed to writing.

## Conflict of Interest Statement

None declared.

## Supplementary Material

Supplementary Data

## Supporting Information

The following additional information is available in the online version of this article —

 **Figure 1**. Simplified schematic of non-spatial simulation. Each simulation contains a fixed number of patches that in the final time step all contain suitable habitat and correspond to observed habitats. These habitats have varying ages, and the simulation begins when only the oldest habitat was in existence. As the simulation progresses, additional habitats appear. Plants colonize patches with suitable habitat according to a random probability that is fixed throughout the simulation. Plants grow, reproduce and die within habitats according to stage-based demographic models.

 **Figure 2.** Flow chart of non-spatial modelling steps simulating demographic growth within a set of discrete habitat envelopes. Model inputs include demographic field data and three *a* *priori* parameters: colonization probability, habitat failure rate and carrying capacity (white ovals). These data are used to construct transition matrices and a distribution of ages for how long each patch has existed as suitable habitat. In each time step, the model randomly adds seeds to all patches and allows patches with extant habitat to grow according to a stochastic stage-based matrix model (black squares). The distribution of stages within each patch is tracked throughout the model, and can be used in the final time step to calculate the percentage of occupied patches.

 **Figure 3**. Matrix of mean transition values and standard deviations in parentheses based on Gawler’s (1988) 26 observed transitions. All individual matrices are provided in the supplementary materials.

 **Figure 4.** Effect of sample size on estimates of mean occupancy and 95 % confidence intervals for non-spatial metapopulation model. Here, we ran 1000 iterations of the non-spatial model with habitat failure rate at 0.01 and colonization probability at 0.4 and calculated the occupancy rate in the final time step. We then randomly sampled, with replacement, from this set of 1000 iterations, drawing between 2 and 1000 values, as represented on the *x*-axis. From the random sample, we estimated the mean occupancy (centre black line). We also fit a beta-distribution to the sample distribution, and used this beta-distribution to estimate the 95 % confidence interval (upper and lower black lines). Red horizontal lines depict the mean, and upper and lower 95th percentiles drawn from the entire set of 1000 iterations without using a beta-distribution to smooth the data. The vertical dashed line at 15 iterations represents the number of iterations run for each of 400 parameterizations in the study.

 **Figure 5.** Simulated occupancy in a spatial model of *Pedicularis furbishiae* at 149 patches estimated from 20 iteration at each of combination of the parameters *f* and *b*. The fraction of dispersal seedlings that disperse downstream is governed by *f*, whereas *b* is the distance cost for seedlings dispersing to the other side of the river, in units of the scale parameter. Patch occupancy is defined as the number of patches with at least one reproductive adult. The characteristic scale defines the exponential distribution used for seedling dispersal.

 **File 1.** Manual for using R code developed for the metapopulation model of Furbish’s lousewort.

 **File 2.** Zipped folder containing: (A) a sub-directory with input data used for the models, (B) an R Source File, (C) a simple R Script File and (D) the full R script we used for model analyses.
