# Supplementary material for: Combining incidence and demographic modelling approaches to evaluate metapopulation parameters for an endangered riparian plant
Source: AoB Plants. 2016 Jul 11;8:plw044. doi: 10.1093/aobpla/plw044 (PMC4940506; doi:10.1093/aobpla/plw044)
Supplement: Supplementary Data [file supp_8_plw044_index.html]

Combining incidence and demographic modelling approaches to evaluate metapopulation parameters for an endangered riparian plant — Supplementary Data 

# Combining incidence and demographic modelling approaches to evaluate metapopulation parameters for an endangered riparian plant

## Supplementary Data

files

- Supplementary Data - zip file
