# Supplementary material for: Combining incidence and demographic modelling approaches to evaluate metapopulation parameters for an endangered riparian plant
Source: AoB Plants. 2016 Jul 11;8:plw044. doi: 10.1093/aobpla/plw044 (PMC4940506; doi:10.1093/aobpla/plw044)
Supplement: Supplementary Data [file supp_plw044_suppl_data.zip › aobplants-15298-s01.docx]

**Supplementary Figure 1.** Simplified schematic of non-spatial simulation. Each simulation contains a fixed number of patches that in the final time step all contain suitable habitat and correspond to observed habitats. These habitats have varying ages, and the simulation begins when only the oldest habitat was in existence. As the simulation progresses, additional habitats appear. Plants colonize patches with suitable habitat according to a random probability that is fixed throughout the simulation. Plants grow, reproduce, and die within habitats according to stage-based demographic models.


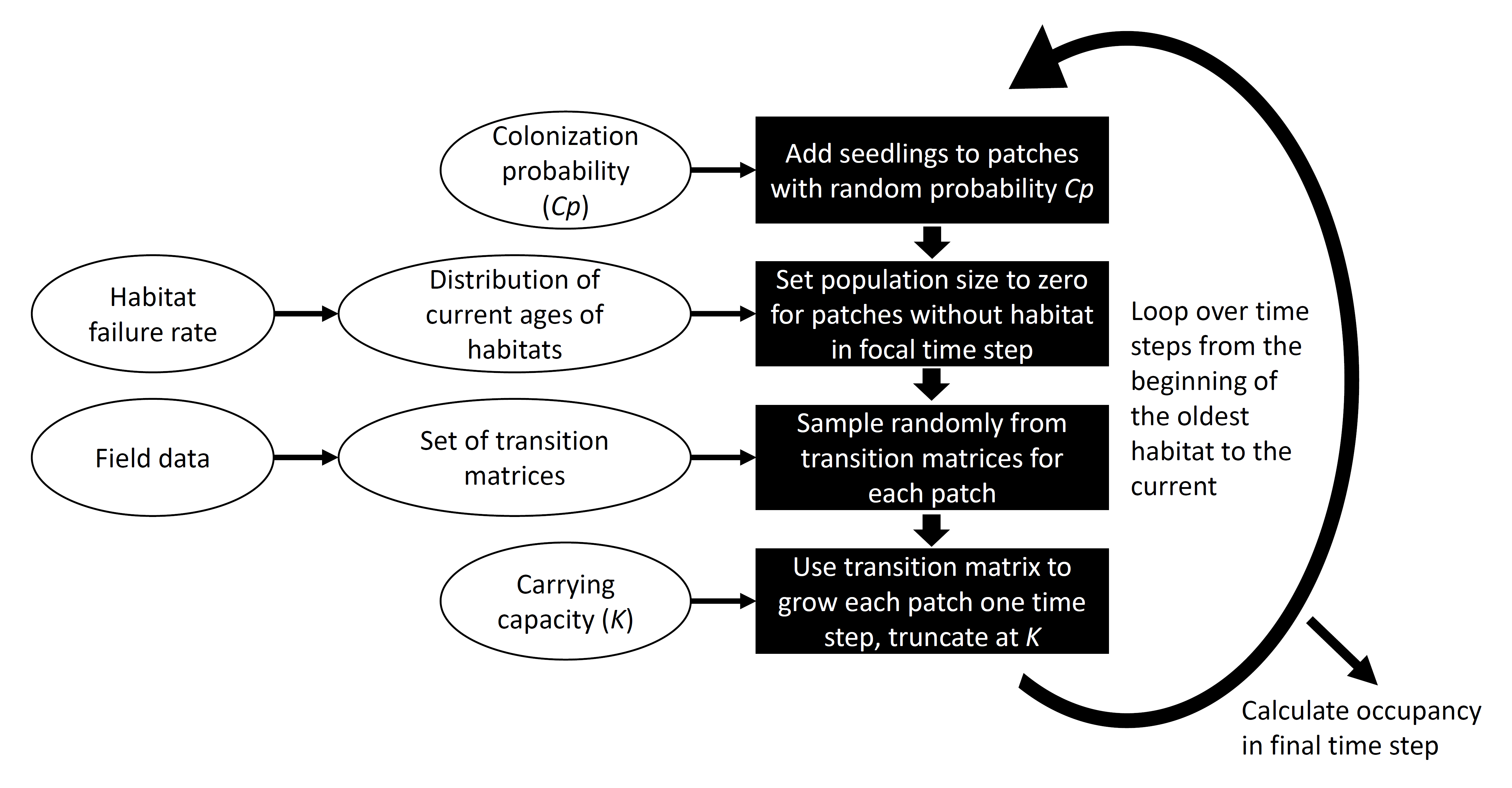


**Supplementary Figure 2.** Flow chart of non-spatial modeling steps simulating demographic growth within a set of discrete habitat envelopes. Model inputs include demographic field data and three a-priori parameters: colonization probability, habitat failure rate, and carrying capacity (white ovals). These data are used to construct transition matrices and a distribution of ages for how long each patch has existed as suitable habitat. In each time step, the model randomly adds seeds to all patches and allows patches with extant habitat to grow according to a stochastic stage-based matrix model (black squares). The distribution of stages within each patch is tracked throughout the model, and can be used in the final time step to calculate the percentage of occupied patches.

**Supplementary Figure 3.** Matrix of mean transition values and standard deviations in parentheses based on Gawler’s (1988) 26 observed transitions. All individual matrices are provided in the supplementary materials.


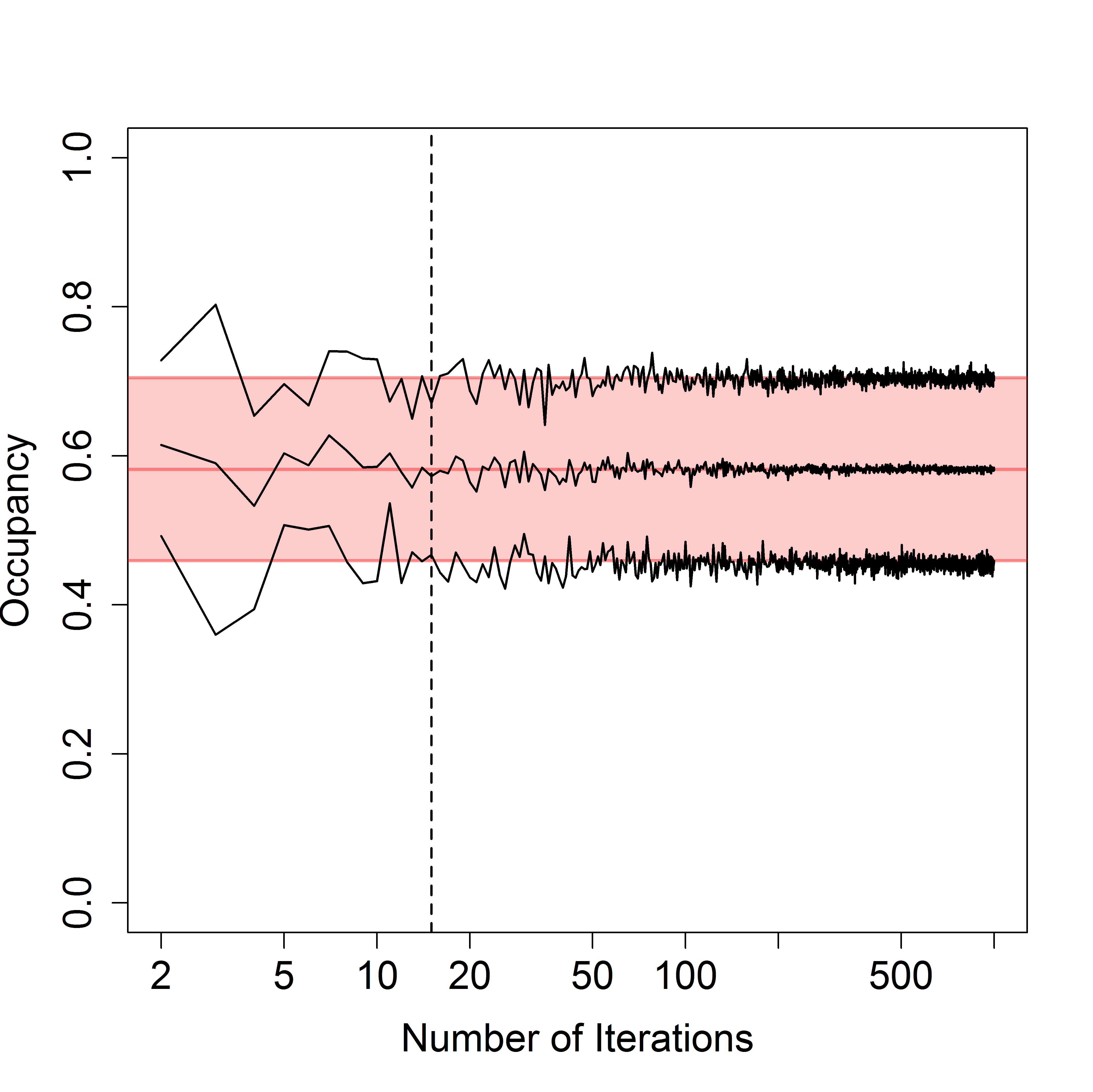


**Supplementary Figure 4.** Effect of sample size on estimates of mean occupancy and 95% confidence intervals for non-spatial metapopulation model. Here, we ran 1000 iterations of the non-spatial model with habitat failure rate at 0.01 and colonization probability at 0.4 and calculated the occupancy rate in the final time step. We then randomly sampled, with replacement, from this set of 1000 iterations, drawing between 2 and 1000 values, as represented on the x-axis. From the random sample, we estimated the mean occupancy (center black line). We also fit a beta distribution to the sample distribution, and used this beta distribution to estimate the 95% confidence interval (upper and lower black lines). Red horizontal lines depict the mean, and upper and lower 95^th^ percentiles drawn from the entire set of 1000 iterations without using a beta distribution to smooth the data. The vertical dashed line at 15 iterations represents the number of iterations run for each of 400 parameterizations in the study.


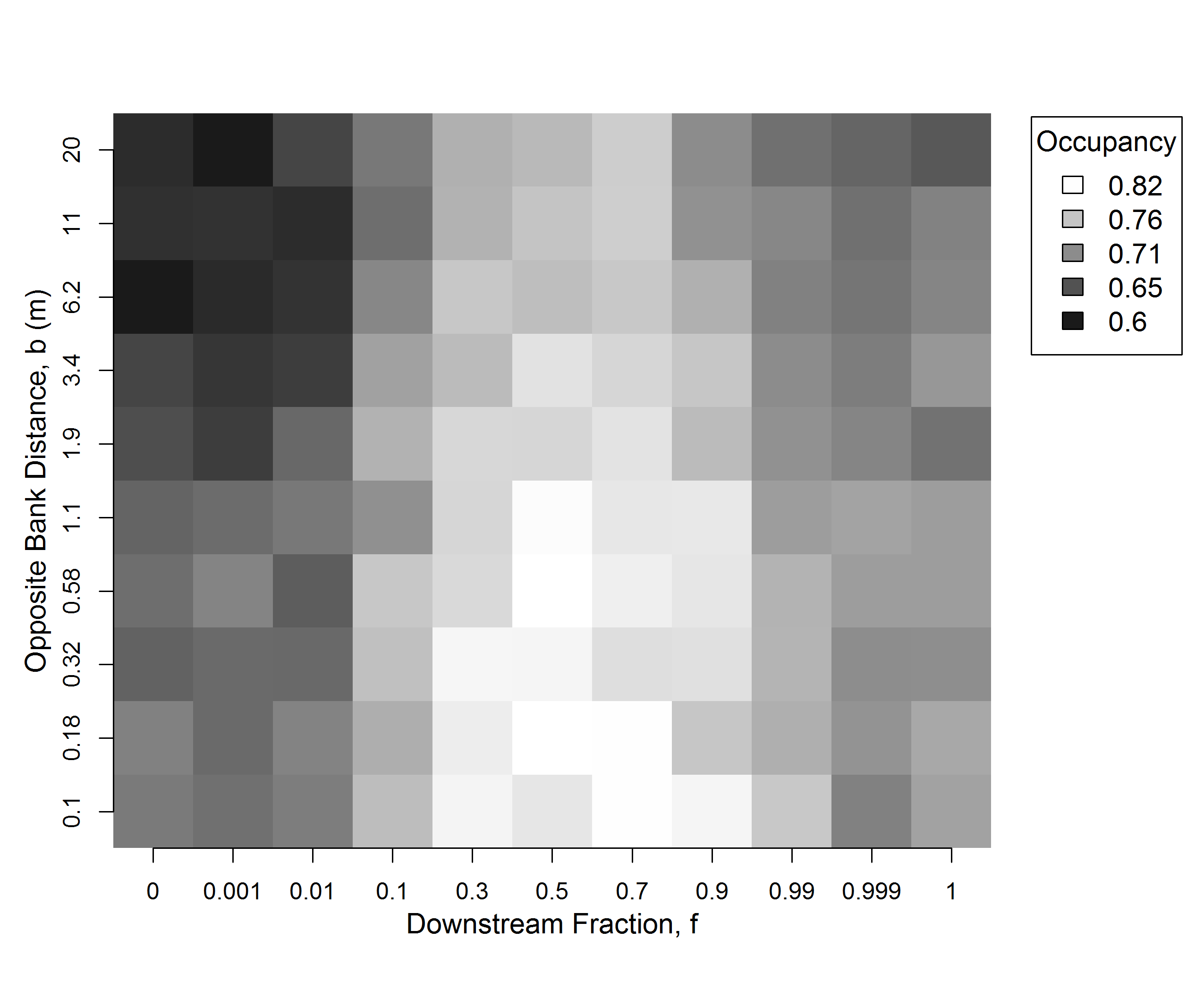
 **Supplementary Figure 5.** Simulated occupancy in a spatial model of *Pedicularis furbishiae* at 149 patches estimated from 20 iteration at each of combination of the parameters *f* and *b.* The fraction of dispersal seedlings that disperse downstream is governed by *f,* whereas *b* is the distance cost for seedlings dispersing to the other side of the river, in units of the scale parameter. Patch occupancy is defined as the number of patches with at least one reproductive adult. The characteristic scale defines the exponential distribution used for seedling dispersal.
