# Supplementary material for: Combining incidence and demographic modelling approaches to evaluate metapopulation parameters for an endangered riparian plant
Source: AoB Plants. 2016 Jul 11;8:plw044. doi: 10.1093/aobpla/plw044 (PMC4940506; doi:10.1093/aobpla/plw044)
Supplement: Supplementary Data [file supp_plw044_suppl_data.zip › aobplants-15298-s03.docx]

Manual for Using R Code Developed for

Metapopulation Model of Furbish’s Lousewort

February, 2016

Contents

[Introduction 1](#_Toc444428421)

[nonspatial.model() 3](#_Toc444428422)

[simple.nonspatial.model() 3](#_Toc444428423)

[spatial.model() 4](#_Toc444428424)

[year.step() 5](#_Toc444428425)

[hab.age.function() 5](#_Toc444428426)

[cP.function() 6](#_Toc444428427)

[get.dispersal.mat() 6](#_Toc444428428)

[get.linear.order.from.XY() 6](#_Toc444428429)

[References 7](#_Toc444428430)

# Introduction

This document describes use of the code written in R (3.1.0) for the manuscript on Furbish’s Lousewort metapopulations (2016). Supplements for this manuscript include a zipped file (“Furbish_Rcode.zip”) containing: a subdirectory with input data used for the model, an R Source File (“Furbish_source.R”), a simple R Script File (“Fubish_tutorialscript.R”), and the full R script we used for model analysis (“Furbish_script.R”). You must unzip the folder before running.

The input data subdirectory contains transition matrices (in the subfolder “T_matrices”) and fecundity variance table (“F_variance.csv”) based on Gawler (1988). This folder also contains data on patches and populations to be used for modeling. The population data provided are simulated and do not represent the actual observations of plant locations or abundances, because Furbish’s lousewort is a federally endangered species and we do not have permission to release these data.

To run the models from our manuscript, you must first download and install the freely available R software (<https://www.r-project.org/>). You can then open the provided simple Script File in R and follow along through the script using the comments (everything after a “#”) to guide you. The simple script parallels the full study script, divided into 4 main sections. In section A, you set up the workspace, which requires downloading necessary R packages, reading in the Source File, and loading the input data. In section B, you run the non-spatial model. In section C, you run the spatial model.

The Source File contains all of the functions which the Script File uses. You may open the Source File to see all of the functions and what their arguments are, but you do not need to. For most of the functions provided, if you enter the name into the R console, the entire function will print out, including descriptions of the required arguments at the beginning (top) of printout.

In the remainder of this document, we provide cursory descriptions of the primary functions developed for these models along with the arguments (aka model parameters) required when running the functions. For full descriptions of the models, please see full manuscript (2016). Several of the functions use the same argument names, and we have included these arguments in Table 1, below.

Table 1. Arguments used by multiple functions.

| Argument Name | Description |
| --- | --- |
| yearly.failure | **Habitat Failure Probability.** The probability that a suitable habitat patch will become unsuitable in any given year. |
| K | **Carrying Capacity.** For the nonspatial model, this is a single number. For the spatial model, this may be either a single number or a vector of the same length as the number of patches. K gives the carrying capacity of each patch as measured by the number of flowering individuals. If a single number, all patches have the same carrying capacity. Population size of flowering individuals will be truncated at K, with proportionate decreases in all stages. Thus, if a matrix multiplication yields 1000 non-flowering and 200 flowering individuals when K is set to 100, then after truncating there will be 500 non-flowering and 100 flowering individuals. |
| T.matrices | **Stage-Based Transition Matrices.** Three-dimensional array of observed transition matrices. The first dimension is the number of transition matrices (e.g. how many year/plot transitions were observed). The second and third dimensions are the number of stages. The demographic growth portions of the models randomly select from among these matrices in each time step. |
| F.vars | **Variance in observed fecundity.** Array of fecundity variance matrices, with the same dimensions as T.matrices |
| youngest.flower.stage | **Stage of flowering.** The index position of the stage at which flowering begins, used to truncate populations at carrying capacity and for the purpose of determining occupancy based on flowering individuals. |
| num.habitats | The number of habitat patches to simulate in model. Only applies to non-spatial models and associated functions. |
| cluster.nodes | (Optional) The number of cores to use in parallel on a cluster set up by the snow package (automated internally). Note that progress reports are suppressed when cluster.nodes > 1. It is not recommended that you run more parallel processes than the number of cores on your computer. Note, if you set cluster.nodes > 1, then there will be one more level of hierarchy in the output list, with the number of items in the top level equal to cluster.nodes. It is possible that on versions of R other than 3.1.0, there will be issues when running parallel processing. |
| source.file: | A character giving the path and name of this source file. E.g. "c:/Furbish/Metapop_source.R." Only necessary if cluster.nodes > 1. |

# nonspatial.model()

**Description**: Runs one iteration of nonspatial model simulating historic metapopulation dynamics based on a propagule-rain model of colonization. Generates a distribution of ages of habitat within patches, then projects growth in habitat patches from age of oldest habitat patch to current year. Outputs a matrix for stage distribution of plants in each habitat patch. This is the inverse modeling (Railsback and Grimm 2011) approach described in main manuscript (2016).

**Arguments described in table 1**: num.habitats, yearly.failure, T.matrices, F.vars, K, youngest.flower.stage,

Table 2. Additional arguments specific to the nonspatial.model().

| Argument Name | Description |
| --- | --- |
| cP | **Colonization probability.** The annual probability that a seed from outside the patch will arrive at the patch and establish as a seedling. |
| truncate | The maximum time back into the past to start the model. When yearly.failure is very low, randomly generated habitat ages within this model will be very old, which will take a long time to run. For example, when yearly.failure is set to 0.001, a bit more than 10% of the habitats will be older than 2000 years. However, if truncate is set to 500, then the maximum habitat age will be 500 years. |
| col.as.binom | Whether colonization should be binomial or poisson process (e.g. only one seedling per patch per year allowed, or multiple allowed). |

**Output**: A matrix with patch habitats as rows and stages as columns giving the distribution of individual stages within each patch in the final year of the model run.

# simple.nonspatial.model()

**Description**: Runs one iteration of forward-looking nonspatial model simulating metapopulation dynamic based on a propagule-rain model of colonization. Begins with no plants in the model. Outputs a matrix for stage distribution of plants in each habitat patch.

**Arguments described in tables 1 & 2**: num.habitats, yearly.failure, T.matrices, F.vars, K, youngest.flower.stage,cP, col.as.binom

**Output**: A matrix with patch habitats as rows and stages as columns giving the distribution of individual stages within each patch in the final year of the model run.

# spatial.model()

**Description**: Projects spatially explicity metapopulation model along a river with dispersal. Primary inputs are patch characteristics, initial conditions and parameters on habitat turnover, dispersal, and demography. Primary outputs are annual subpopulation counts.

**Arguments described in Table 1**: yearly.failure, T.matrices, F.vars, K, youngest.flower.stage.

**Table 3**. Additional arguments specific to the spatial.model().

| Argument Name | Description |
| --- | --- |
| dispersing.ratio | **Dispersal recruitment (*r*).** The ratio of additional recruitment outside of the patch boundaries relative to internal recruitment. For example, when r = 0.1, for every 10 seedlings established at the end of a demographic run within patch i, we would allow 1 additional seedling to disperse from patch i to another point along the river. |
| scale.parameter | **Scale Parameter (1/*τ*).** The characteristic scale (in same units as location data) of the exponential distribution used for calculating dispersal probability |
| opp.bank.dist | **Cost factor for dispersing across the river (*b*).** A number in units of the scale parameter giving the distance cost to dispersal across the river to the opposite bank. When opp.bank.dist is set to 1, that means we add an extra distance equal to scale.parameter between all patches on opposite banks from each other prior to estimating dispersal probabilities. |
| downstream.percent | **Fraction of downstream dispersal (*f*).** A number between 0 and 1 giving percent of dispersal that goes downstream. |
| years | The number of years (or time steps) to run the model forward. |
| patch.stages.0 | A matrix with Patch as rows and Stages as columns giving the initial abundance of each stage in each patch. |
| patch.hab | Vector of the same length as the number of patches consisting of 1s and 0s indicating whether each patch initially contains suitable (1) or unsuitable (0) habitat. |
| patch.position | Vector of the same length as the number of patches giving the 1-dimensional coordinate of the center of each patch along the river geometry. For example, (0,100,1300) could mean the three patches occur at 0m, 100m and 1300m from the end of the river. |
| patch.length | Vector of the same length as the number of patches giving the size of each patch (length along river geometry). |
| patch.side | Vector of the same length as the number of patches indicating which bank each patch is on. Should have just two levels (North/South, 1/2, Left/Right...) |
| patch1.headwater. | This is a logical value (TRUE or FALSE) indicating the direction of flow in the river. According to patch.position, is the point with the smallest value (e.g. 0) closer to the headwater (patch1.headwater = TRUE), or closer to the river mouth (patch1.headwater = FALSE). |

**Output**: spatial.model() will output a “list” consisting of three items: occupancy.mat, habitat.mat, and colonize.mat. Each of these items is a matrix with patches on rows and simulation years as columns.

occupancy.mat: The total number of flowering stems in each patch in each year.

habitat.mat: Whether each patch was suitable (1) or unsuitable (0) habitat in each year.

colonize.mat: Whether each patch was suitable (1) or unsuitable (0) habitat in each year.

For example, if you made the following call (including all parameters in “…”)

projection.out <- spatial.model(dispersing.ratio = 1, scale.parameter = 1, …)

Then, if you type “projection.out$occupancy.mat” into the R console, you will see the full occupancy matrix.

# year.step()

**Description**: Grows a single subpopulation forward one time step, including stochastic processes. Uses initial stage distribution and demographic parameters to produce stage distribution for the next year.

**Arguments described in Table 1**: T.matrices, F.vars, K, youngest.flower.stage.

**Table 4**. Additional arguments specific to year.step().

| Argument Name | Description |
| --- | --- |
| n0 | **Initial stage distribution.** A vector of the same length as the number of stages giving the initial number of individuals in each stage. |

**Output**: A vector of the stage distribution after applying transition matrices.

# hab.age.function()

**Description**: Generates a distribution of habitat ages by drawing from an exponential distribution.

**Arguments described in Table 1**: num.habitats, yearly.failure, truncate.

**Output**: A matrix with dimensions patches as rows and years as columns, giving a "1" for every year that the focal patch supported habitat, and a "0" for years that it did not support habitat. In the earliest year (first column), only a single patch will contain habitat, and in the latest year (last column), all patches will contain habitat. Patches only switch from non-habitat to habitat, never the other way around.

# cP.function()

**Description**: Steps through nonspatial model for all given colonization rates, outputs habitat occupancies rates in current year for each colonization probability. Occupancies are counted only for populations with plants at or above flowering stage.

**Arguments described in Table 1**: yearly.failure, youngest.flower.stage, T.matrices, F.vars, K, truncate, cluster.nodes, source.file.

**Table 4**. Additional arguments specific to year.step().

| Argument Name | Description |
| --- | --- |
| cP.vec | A vector of colonization probabilities to step through. |

**Output**: Matrix where the first row gives the focal colonization probability, the second row gives the number of occupied patches, and the columns represent time steps. Occupancies are counted only for populations with plants at or above flowering stage.

# get.dispersal.mat()

**Description**: Calculates the probability for a dispersing individual from patch i to land within patch j. We do not allow dispersal from patch i to patch i (in other words, diagonal elements in output matrix are 0). Assumes linear, one-dimensional geometry (e.g. a river).

**Arguments described in Table 3 (spatial model table)**: scale.parameter, patch.length, downstream.percent, patch1.headwater.

**Table 4**. Additional arguments specific to year.step().

| Argument Name | Description |
| --- | --- |
| distance.mat | Matrix of all pairwise distances between patches, with both rows and columns being the same set of patches. Both the upper and lower triangles should be filled. |

**Output**: A square matrix giving the probability of dispersing from patch i to j.

# get.linear.order.from.XY()

**Description**: Given a series of (x,y) coordinates, this function organizes them so that they form the simplest series of line segments, e.g. assuming they are points along a river. The output is the order of points and requires user interface with the plotting device. Note, that if the river doubles back on itself and there are few points in this region, it is possible for undesirable ordering of points to occur.

**Table 5**. Arguments specific to get.linear.order.from.XY().

| Argument Name | Description |
| --- | --- |
| patch.coords | A two-column matrix where rows are points and columns are X and Y coordinates. |

**Output**: When this function is run, a plot should appear displaying the points. The user must then place cursor closest to the first point along the river, and click. The function will then plot a series of line segments connecting the points along the linear geometry of the river. The user will then be asked if any points need correcting, at which point, if two points are out of order, you click on the two points in order, then be prompted to identify the first point in the remaining stretch of river. This will continue iteratively until hopefully everything looks right. The output is a vector giving the ordering of points along the river.

# References

Gawler, S.C. 1988. Disturbance-mediated population dynamics of Pedicularis furbishiae S. Wats., a rare riparian endemic. Dissertation. University of Wisconsin, Madison, Wisconsin.

Railsback, S.F. and Grimm, V., 2011. Agent-based and individual-based modeling: a practical introduction. Princeton university press.

Jochen Knaus (2013). snowfall: Easier cluster computing (based on snow).. R package version 1.84-6. <http://CRAN.R-project.org/package=snowfall>.

Lemon, J. (2006) Plotrix: a package in the red light district of R. R-News, 6(4): 8-12.

Luke Tierney, A. J. Rossini, Na Li and H. Sevcikova (2013). snow: Simple Network of Workstations. R package version 0.3-13. <http://CRAN.R-project.org/package=snow>.

Stubben, C.J. and Milligan, B.G. 2007. Estimating and Analyzing Demographic Models Using the popbio Package in R. Journal of Statistical Software 22:11.
