# Supplementary material for: Combining incidence and demographic modelling approaches to evaluate metapopulation parameters for an endangered riparian plant
Source: AoB Plants. 2016 Jul 11;8:plw044. doi: 10.1093/aobpla/plw044 (PMC4940506; doi:10.1093/aobpla/plw044)
Supplement: Supplementary Data [file supp_plw044_suppl_data.zip › aobplants-15298-s04.docx]

# OPEN ACCESS – RESEARCH ARTICLE

# Combining incidence and demographic modeling approaches to evaluate metapopulation parameters for an endangered riparian plant.

### Noah D. Charney* and Sydne Record

### Bryn Mawr College, Department of Biology, 101 North Merion Ave., Bryn Mawr, PA 19010

*Corresponding author

Corresponding author’s e-mail address: Noah@alumni.amherst.edu

Running head: Modeling Furbish’s lousewort metapopulation
